# Quantitative Prediction of Acid Value of Camellia Seed Oil Based on Hyperspectral Imaging Technology Fusing Spectral and Image Features

**DOI:** 10.3390/foods13203249

**Published:** 2024-10-12

**Authors:** Yuqi Gu, Lifang Shi, Jianhua Wu, Sheng Hu, Yuqian Shang, Muhammad Hassan, Chao Zhao

**Affiliations:** 1 College of Optical, Mechanical and Electrical Engineering, Zhejiang A&F University, Hangzhou 311300, China; guyuqi@zafu.edu.cn (Y.G.); shilifang@stu.zafu.edu.cn (L.S.); 2 Panzhihua Academy of Agriculture and Forestry Sciences, Panzhihua 617061, China; jhuawu2024@163.com; 3 National Engineering Technology Research Center of Forestry and Grassland Machinery for Hilly and Mountainous Areas, State Forestry and Grassland Administration, Hangzhou 311300, China; hs15057087528@163.com; 4 Key Laboratory of Agricultural Equipment for Hilly and Mountainous Areas in Southeastern China, Ministry of Agriculture and Rural Affairs, Hangzhou 311300, China; shangyuqian@stu.zafu.edu.cn; 5 U.S.-Pakistan Center for Advanced Studies in Energy (USPCAS-E), National University of Sciences and Technology, Islamabad 44000, Pakistan; hassan@uspcase.nust.edu.pk

**Keywords:** camellia seed oil, acid value, hyperspectral imaging technology, characteristic wavelength, fusing spectral and image features

## Abstract

Acid value (AV) serves as an important indicator to assess the quality of oil, which can be used to judge the deterioration of edible oil. In order to realize the quantitative prediction of the AV of camellia seed oil, which was made from camellia oleifolia, hyperspectral data of 168 camellia seed oil samples were collected using a hyperspectral imaging system, which were related to their AV content measured via classical chemical titration. On the basis of hyperspectral full wavelengths, characteristic wavelengths, and fusing spectral and image features, the quantitative prediction AV models for camellia seed oil were established. The results demonstrating the 2Der-SPA-GLCM-PLSR model fusing spectral and image features stood out as the optimal choices for the AV prediction of camellia seed oil, with the correlation coefficient of calibration set ($R_{c}^{2}$) and the correlation coefficient of prediction set ($R_{p}^{2}$) at 0.9698 and 0.9581, respectively. Compared with those of 2Der-SPA-PLSR, the $R_{c}^{2}$ and $R_{p}^{2}$ were improved by 2.11% and 2.57%, respectively. Compared with those of 2Der-PLSR, the $R_{c}^{2}$ and $R_{p}^{2}$ were improved by 5.02% and 5.31%, respectively. Compared with the model based on original spectrum, the $R_{c}^{2}$ and $R_{p}^{2}$ were improved by 32.63% and 40.11%, respectively. After spectral preprocessing, characteristic wavelength selection, and fusing spectral and image features, the correlation coefficient of the optimal AV prediction model was continuously improved, while the root mean square error was continuously decreased. The research demonstrated that hyperspectral imaging technology could precisely and quantitatively predict the AV of camellia seed oil and also provide a new environmental method for detecting the AV of other edible oils, which is conducive to sustainable development.

## 1. Introduction

Lipids in edible oil are one of essential macronutrients that the human body must ingest [1]. They are not only the energy source of the human body, but also contain many nutrients needed by the human body, such as polyunsaturated fatty acids, tocopherols, phytosterols, squalene, and so on [2,3]. However, edible oil can be hydrolyzed into free fatty acids and cause rancidity due to the effects of heat, light, oxygen in the air, moisture in the oil, and enzymes during transportation and storage [4,5]. Consuming rancid oils can harm human health, causing gastrointestinal disorders, diarrhea, and even damaging liver and kidney function. Long-term consumption of rancid oils can disrupt the balance of human functions and seriously damage human health [5]. The quality of edible oil is related to human health, safety, and development [6,7]. Acid value (AV) is a quality indicator to assess the content of free fatty acid in edible oil, and AV is a mandatory inspection item of edible oil stipulated in the national food safety standard, which can measure the deterioration degree of oil [8].

At present, the main methods for determining AV include cold solvent indicator titration, cold solvent automatic potentiometric titration, and hot ethanol indicator titration [9]. Although these methods measure accurately, they have high detection costs, cumbersome operations, sample destruction, and high reagent consumption, making it difficult to meet the requirements of modern society for rapid, real-time, and on-site testing of large-scale industrial food safety detection. In recent years, a rapid detection method of AV has emerged. Its principle is to detect the AV of edible oil using the color change in test paper after the reaction between a free fatty acid and composite indicator, so it is still impossible to avoid using chemical reagents, and the detection accuracy needs to be further improved [9].

Hyperspectral imaging technology uses a large number of high-precision image data in narrow and continuous wavelengths and reflects the relevant information of the tested sample through the subtle changes in spectral information [10]. It is a photoelectric detection technology that has developed rapidly in recent years [11]. Hyperspectral imaging technology has advantages such as nondestructive testing, rapid testing speed, low cost, green and low-carbon, and it can achieve synchronous measurement of multiple indicators [12]. Various spectroscopy technology have been applied in various fields such as agriculture [13,14], food [15,16], medical care [17], and animal husbandry [18]. Many scholars have carried out a series of studies on the quality detection of various foods using hyperspectral imaging technology [19,20]. Hui Z et al. identified the quality of oil products when comparing the differences in hyperspectral transmission curves of different oils within a specific wavelength range [21]. Chengqian J et al. employed near-infrared hyperspectral imaging technology to detect the moisture content of soybean and found that the Normalize-SPA-PCR model had optimal performance and could achieve visualization of soybean moisture content [22]. Hongju H et al. detected the change in the acid value of pork through near-infrared hyperspectral imaging technology. The study showed that the RAW-RC-PLS model had the best effect in predicting the acid value of pork and could realize rapid and nondestructive evaluation of the acid value of pork [23]. Hongping Z et al. applied a hyperspectral imaging system to determine the oil content of camellia seed oil. Comparing the PLSR models of Spectral Set I (400–1000 nm) and Spectral Set II (900–1700 nm), it was found that the model established by Spectral Set II had higher accuracy and a wavelength range of 900–1700 nm and was more suitable for oil content detection [24]. Da Silva Medeiros et al. applied hyperspectral imaging technology to detect the fatty acid of rapeseeds, and the PLS-DA model could accurately classify rapeseeds [25]. Malavi et al. established PLS-DA and PLS models using hyperspectral imaging technology to classify and quantify adulterated olive oil. The results indicated that the PLS model had the best performance, with a high predicted correlation, which could detect adulterated olive oil [26]. Van Haute et al. could quickly and accurately predict the content of essential oil (EO) in spearmint based on hyperspectral imaging technology, and the model established through combination of partial least squares regression (PLSR) and multilayer perceptron (MLP) after pretreatment of standard normal variable (SNV) had the best predictive performance [27].

Camellia seed oil is one of the oldest woody edible plant oils unique to China, which contains various pharmacological active ingredients and enjoys the reputation of “Eastern Olive Oil” [28]. As a high value-added product, the rancidity of camellia seed oil can affect its quality and pose a threat to food health and safety. So, the quality determination of camellia seed oil has become an urgent problem. This study focused on camellia seed oil and adopted hyperspectral imaging technology to collect the hyperspectral data of camellia seed oil samples. Meanwhile, the AV of camellia seed oil was measured using classical chemical titration. The obtained hyperspectral data were correlated with the measured AV data. Different preprocessing methods were used, such as selecting suitable characteristic wavelengths, fusing spectral and image features, and combining principal component regression (PCR) and the partial least squares regression (PLSR) approach to establish a quantitative prediction model for the AV of camellia seed oil. This study could provide methodological reference and data support for online, rapid, and noncontact measurement of the AV in edible oil.

## 2. Materials and Methods

### 2.1. Sample Collection

Camellia seed oil samples were made from camellia oleifolia and gathered from three different production areas including Qiandaohu in Zhejiang Province, Qiyunshan in Jiangxi Province, and Sanjiang in Guangxi Province from July and August 2022. From each production area, 56 samples and an aggregate of 168 samples were obtained. The samples were stored in a refrigerator (4 °C) for future use after collection.

### 2.2. Determination of AV in Camellia Seed Oil

The AV of camellia seed oil was determined via the hot ethanol method according to ISO 660: 2020—“Animal and Vegetable Fats and Oils—Determination of Acid Value and Acidity” [29]. The specific process was as follows: 50 mL of 95% ethanol solution and 2 g of oil sample were weighed with a balance, then the weighed ethanol and oil were poured into a 100 mL beaker, and 3~4 drops of 10 g/L phenolphthalein indicator were added. We heated the beaker using a heat-collecting constant-temperature heating magnetic stirrer (DF-101S, Hebei Tianqi Xingzi Testing Equipment Co., Ltd., Cangzhou, China) until the liquid was boiling. Finally, we titrated 0.1 mol/L potassium hydroxide solution with a microtiter tube until it turned red. The consumption volume of potassium hydroxide solution V was calibrated. The formula for calculating the AV in camellia seed oil is shown in Equation (1).

(1) AV=56.1×C×V

where C is the exact concentration of the standard potassium hydroxide solution used (mol/L); V is the volume of the standard potassium hydroxide solution used (mL); and m is the mass of the test portion (g). The AV of each sample was measured three times and the averaged value was reported.

### 2.3. Hyperspectral Imaging System and Data Acquisition

#### 2.3.1. Hyperspectral Imaging System

**Figure 1:**
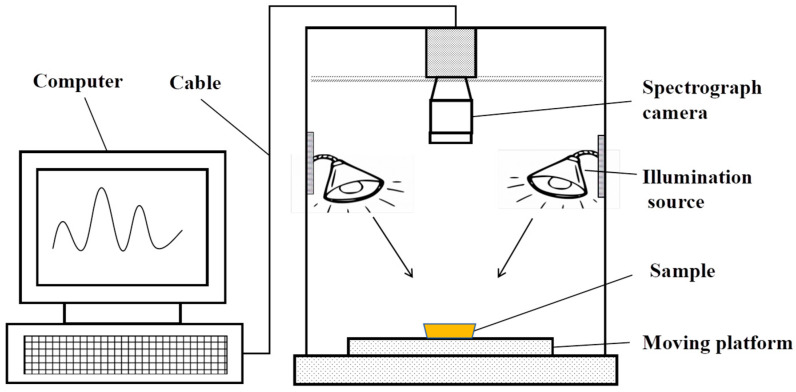
Schematic diagram of hyperspectral imaging system.

A hyperspectral imaging instrument with a wavelength of 870~1720 nm and a resolution of 5 nm (GaiaField-N17E, Shuangli Hepu Technology Co., Ltd., Wuxi, China) was employed in the hyperspectral imaging system, and it was equipped with an electronically controlled moving platform (PSA200-11-X, Li Zhuo Hanguang Instrument Co., Ltd., Tongzhou, Beijing, China) and four 150 W halogen tungsten lamp light sources (ModelsXC-130, Shanghai Jiepu Technology Co., Ltd., Xuhui, Shanghai, China), imaging lens, a 270 × 310 pixel camera, computer, and monitor. The schematic diagram of the hyperspectral imaging system is shown in Figure 1.

#### 2.3.2. Hyperspectral Data Collection, Information Extraction, and Sample Division

**Figure 2:**
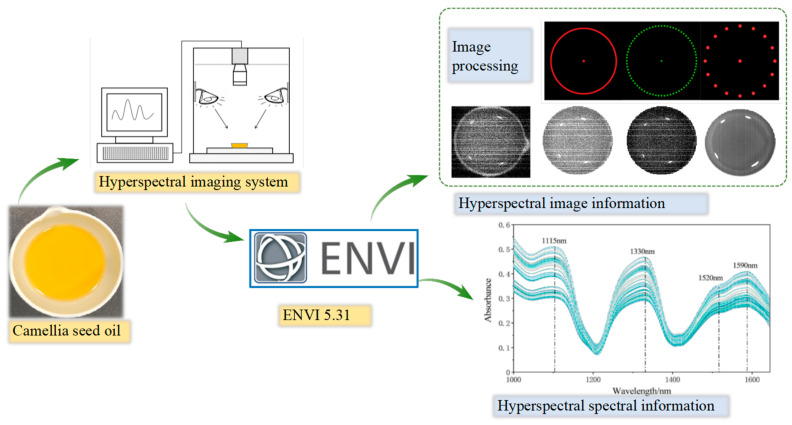
Spectrum information acquisition and extraction process.

The process of collecting the hyperspectral data of the camellia seed oil was as follows: 10 ± 0.05 g camellia seed oil was added into a 30 mL ceramic evaporating dish, then the evaporating dish was placed on the workbench for data collection. Each picture was scanned with 4 samples, three times repeatedly, and the average of three spectral data was taken for reflectivity calibration analysis. ENVI 5.31 was applied to process the hyperspectral image data, including cropping, background segmentation, image masking, and region of interesting (ROI) extraction (as shown in Figure 2). When extracting the ROI, we took the camellia seed oil region in each image as the ROI, extracted the spectral reflectance of all pixels, and calculated the average spectral reflectance. In the process of extracting hyperspectral spectral and image information, 15 abnormal samples were removed and the remaining 153 samples were subjected to subsequent experiments. Kennard–Stone (K-S) classification method was applied to classify the samples according to the Euclidean distance of spectral interval, in which the samples with the largest distance were divided into a calibration set, and the rest were divided into a prediction set. The advantage of this method was that the samples in the calibration set could be evenly distributed according to spatial distance. The samples were divided into the calibration set and prediction set in a 3:2 ratio according to previous study [16,28]. Therefore, the calibration set and prediction set had 92 and 61 samples (as shown in Table 1), respectively.

### 2.4. Processing and Analysis of Hyperspectral Data

#### 2.4.1. Spectral Preprocessing

In order to reduce the interference of external factors and enhance the accuracy and stability of the prediction model, the acquired spectral data were preprocessed. The preprocessing measures employed in this study included Savitzky–Golay smoothing (SG), normalization, standard normal variate (SNV), first derivative (1Der), second derivative (2Der), etc.

#### 2.4.2. Characteristic Wavelengths Selection

In order to reduce the computational complexity and time during modeling, it was necessary to select characteristic wavelengths from full wavelengths. The characteristic wavelengths selection methods used in this study were successive projection algorithm (SPA) and competitive adaptive reweighting algorithm (CARS), according to previous study [22,30].

#### 2.4.3. Image Texture Feature Analysis

The quality of camellia seed oil included not only the internal quality, but also the external quality. Hyperspectral imaging technology could obtain spectral and image information at any wavelength. The difference in image characteristics was also one of the manifestations of camellia seed oil quality [31]. The gray level co-occurrence matrix (GLCM) is a method to describe texture features according to the sample image information, which could describe the related information of image gray levels in adjacent intervals, directions, and changing ranges [31]. The definition of GLCM is expressed as p(i,j/d,θ), the probability that the gray value is j from a pixel point with gray level i leaving a fixed position (distance d, orientation θ). Inthisstudy,the GLCM was applied to extract image features from images at characteristic wavelengths, and four texture feature parameters including inverse different moment $f_{1}$, angular second moment $f_{2}$, entropy $f_{3}$, and correlation index $f_{4}$ at four directions of 0°, 45°, 90°, and 135° were extracted to describe the image texture difference in the camellia seed oil.

Inverse different moment $f_{1}$ reflects the heterogeneity and complexity of image texture features, and the calculation formula is expressed in Equation (2).

(2) $$f_{1}=\sum_{i=1}^{k}\sum_{j=1}^{k}\frac{p(i,j)}{1+{\left(i-j\right)}^{2}}$$

Angular second moment $f_{2}$ is used to describe the uniformity of image texture distribution, and the calculation formula is expressed in Equation (3).

(3) $$f_{2}=\sum_{i=1}^{k}\sum_{j=1}^{k}{\left[p\left(i,j\right)\right]}^{2}$$

Entropy $f_{3}$ is used to describe the image size. The calculation formula is expressed in Equation (4).

(4) $$f_{3}=-\sum_{i=1}^{k}\sum_{j=1}^{k}p\left(i,j\right)log\left[p(i,j)\right]$$

Correlation $f_{4}$ is used to describe the similarity degree of data information in a row or a column direction of GLCM, which is used as the linear correlation coefficient value of matrix gray level. The calculation formula is expressed in Equation (5).

(5) $$f_{4}=\sum_{i=1}^{k}\sum_{j=1}^{k}\frac{ij*p(i,j)-u_{1}u_{2}}{{\sigma_{1}}^{2}{\sigma_{2}}^{2}}$$

where, $u_{1}=\sum_{i=1}^{k}i\sum_{j=1}^{k}p\left(i,j\right)$, $u_{2}=\sum_{j=1}^{k}j\sum_{i=1}^{k}p\left(i,j\right)$, ${\sigma_{1}}^{2}=\sum_{i=1}^{k}{\left(i-u_{1}\right)}^{2}\sum_{j=1}^{k}p\left(i,j\right)$, and ${\sigma_{2}}^{2}=\sum_{j=1}^{k}{\left(j-u_{2}\right)}^{2}\sum_{i=1}^{k}p\left(i,j\right)$.

### 2.5. Establishment and Evaluation of AV Quantitative Model of Camellia Seed Oil

In this paper, principal component regression (PCR) and partial least squares regression (PLSR) were employed to establish quantitative prediction models for the acid value of camellia seed oil.

- Principal component regression (PCR)

Principal component regression (PCR) uses principal component analysis to decompose spectral matrix A, selects the principal component quantities with higher contribution rates, and then performs multiple linear regression analysis. PCR effectively reduces noise, baseline interference, and improves the stability and accuracy of the model by reintegrating spectral information [32]. The PCR calculation formula is expressed in Equation (6).

(6) $$A=F_{n\times m}\times P_{f\times p}\times E\times f$$

where $F_{n\times m}$, orthogonality between the score matrix of spectral matrix A and the row variables; $P_{f\times p}$, orthogonality between column variables of the load matrix of the principal component; E, residual; and f, number of principal components.

- Partial least squares regression (PLSR)

Partial least squares regression (PLSR) is a statistical method for regression analysis, which is used to model the relationship between dependent variables and multiple independent variables. PLSR combines the characteristics of principal component analysis (PCA) and multiple linear regression and is used to deal with the situation of high collinearity between independent variables. In PLSR, the dependent variable regresses on the composition, not on the original independent variable. The model estimates the coefficient of each component to predict the dependent variable. This method is helpful to reduce multicollinearity and provide more stable and accurate predictions than the traditional multiple linear regression [23,24].

The accuracy of the model was mainly evaluated via the correlation coefficient of the calibration set ($R_{c}^{2}$) and the root mean square error of the calibration set (RMSEC). The prediction ability of the model was evaluated using the correlation coefficient of the prediction set ($R_{p}^{2}$) and the root mean square error of the prediction set (RMSEP). Among them, the higher the $R_{c}^{2}$ and $R_{p}^{2}$ and the lower the RMSEC and RMSEP, the better the regression fitting effect of the model [15].

## 3. Results and Discussion

### 3.1. Determination Results of AV in Camellia Seed Oil

Table 1 shows the AV determination results of the camellia seed oil samples. The maximum AV of camellia seed oil was 1.59 mg/g, the minimum was 0.32 mg/g, and the average was 0.70 mg/g. The AV of camellia seed oil was similar to that of the previous study. According to the previous study, the acid value in rice bran oil ranges from 0.003 to 3.961 mg/g [33]. The acid value in rapeseed oil ranges from 0.15 to 4.15 mg/g, while that in soybean oil ranges from 0.10 to 3.64 mg/g [34]. It is reported that the acid value in olive oil ranges from 0.12 to 1.99 mg/g [20]. The measurement results of the AV showed that the quality of camellia oil was better than that of ordinary vegetable oil and comparable to that of olive oil. It was reported that the average AVs of *C. oleifera*, *C. japonica*, and *C. sinensis* seed oil were 0.3, 1.7, and 0.7 mg/g, respectively [35]. It can be seen that the variety of camellia seed oil and the refining process method had significant influence on the AV of camellia seed oil. All samples were divided into a calibration set and a prediction set with a ratio of 3:2, and the division result is shown in Table 1. From this table, it is obvious that the range of the AV in the prediction set covered the range of that in the calibration set, which met the requirements for constructing hyperspectral modeling and prediction.

### 3.2. Analysis of Hyperspectral Characteristics of Camellia Seed Oil

**Figure 3:**
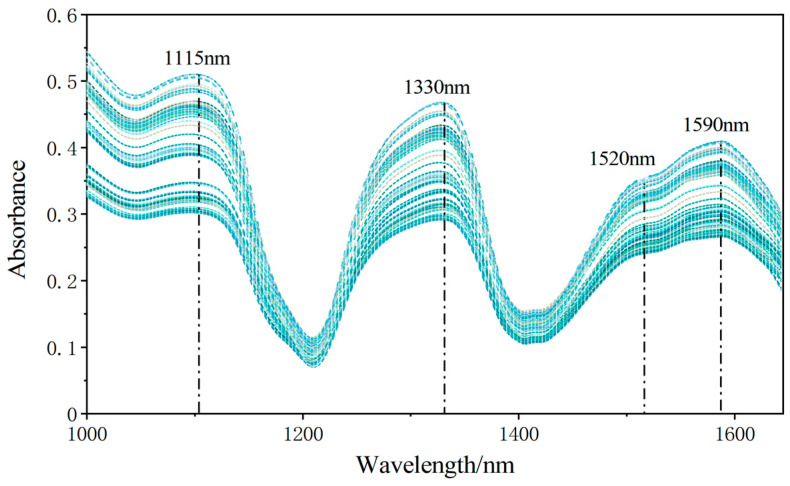
Hyperspectral of camellia seed oil.

The hyperspectral of camellia seed oil is shown in Figure 3. The spectrum of camellia seed oil had three obvious strong absorption peaks at 1115 nm, 1330 nm, and 1590 nm, and a weak absorption peak at 1520 nm. Among them, the strong absorption peaks at 1115 nm indicated the presence of the stretching vibration of the C-H bond. The strong absorption peak at 1330 nm was attributed to the bending vibration of the C-H bond, which was caused by the influence of acid-based compounds. The absorption at 1520 nm was attributed to the bending vibration of the O-H bond. The similar characteristic peaks relating to camellia seed oil were observed in the study of the adulteration level detection of camellia seed oil [28]. It can be seen that the obtained spectrum contained the information needed for modeling construction.

### 3.3. Quantitative Modeling Analysis of Camellia Seed Oil AV Based on Full Wavelengths

The quantitative AV prediction models of camellia seed oil established via PCR and PLSR after different preprocessing methods are shown in Table 2. From the perspective of PCR modeling, the correlation coefficient of the prediction set based on the original spectrum was 0.6962. The predictive performance of the AV model preprocessed using 1Der decreased by 6.97% compared with that based on the original spectrum, which would be caused by the removal of effective information on AV by a derivative. However, after the preprocessing of Normalize, 2Der, and SG, the correlation coefficient of the prediction set of the AV model was obviously improved. Among them, the AV model after 2Der preprocessing had the best performance, with $R_{c}^{2}$ of 0.9198 and $R_{p}^{2}$ of 0.9087. Compared with those of models based on the original spectrum, $R_{c}^{2}$ and $R_{p}^{2}$ were improved by 17.59% and 21.25%, respectively. From the perspective of PLSR modeling, the AV models after 1Der, Baseline, and SG preprocessing showed a modest enhancement in performance, while the AV models employing Normalize and SNV preprocessing exhibited a slight decrease. The AV model after 2Der preprocessing had the best performance, with $R_{c}^{2}$ of 0.9234 and $R_{p}^{2}$ of 0.9098. Compared with those of models based on the original spectrum, $R_{c}^{2}$ and $R_{p}^{2}$ were improved by 19.22% and 22.605%, respectively. In summary, the results of the AV model for camellia seed oil based on full wavelengths suggested that the PLSR model established after 2Der preprocessing (2Der-PLSR) had the best predictive performance.

### 3.4. Quantitative Modeling Analysis of Camellia Seed Oil AV Based on Characteristic Wavelengths

**Figure 4:**
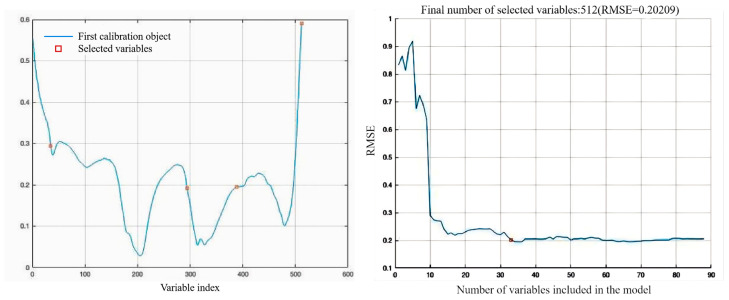
Characteristic wavelengths of camellia oil selected using the SPA method.

In terms of characteristic wavelengths SPA selecting, this study identified four characteristic wavelengths (as shown in Figure 4), namely 1278.78 nm, 1367.98 nm, 1488.66 nm, and 1677.98 nm. The characteristic wavelengths selected using the SPA method accounted for 0.78% of the full wavelengths. A quantitative AV prediction model for camellia seed oil based on these four SPA-selected characteristic wavelengths was established.

**Figure 5:**
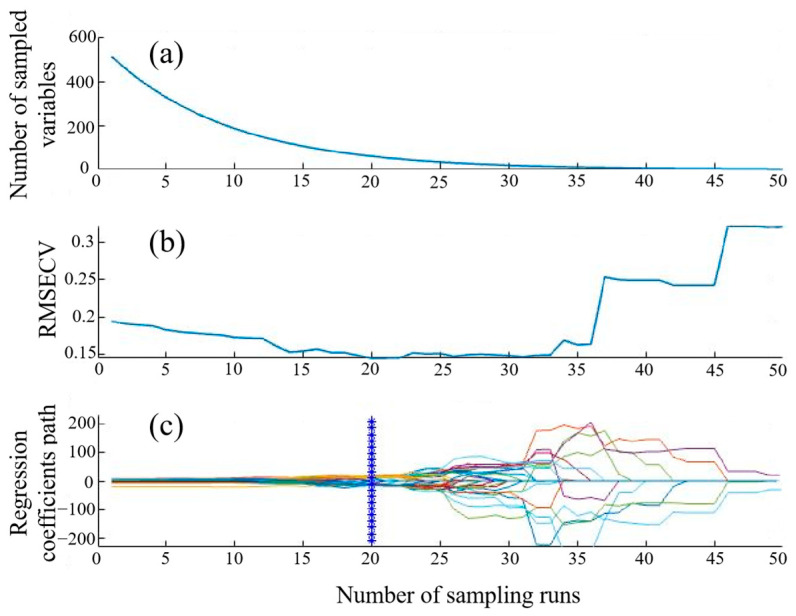
Characteristic wavelengths of camellia oil selected by CARS method. (**a**) Variation trend in the number of variables with the number of samples; (**b**) RMSECV; (**c**) the change process of the regression coefficient of each variable with sampling times (the blue line represents the position with the lowest RMSECV).

In terms of characteristic wavelengths CARS selecting, this study identified 11 characteristic wavelengths (as shown in Figure 5), namely 991.22 nm, 1000.01 nm, 1009.55 nm, 1137.80 nm, 1278.78 nm, 1312.77 nm, 1677.98 nm, 1488.66 nm, and 1598.12 nm, respectively. The characteristic wavelengths selected by CARS method accounted for 2.15% of the full wavelengths. A quantitative AV prediction model for camellia seed oil based on the 11 CARS-selected characteristic wavelengths was established.

The selection results of characteristic wavelengths using the SPA and CARS methods are shown in Table 3. The proportion of characteristic wavelengths selected using SPA in the full wavelengths was 0.78%, and that of those selected using CARS was 2.15%. In spectral processing, SPA and CARS are often applied to select characteristic wavelengths. In hyperspectral modeling of soybean water, 14 and 16 characteristic wavelengths were selected using SPA and CARS, accounting for 6.5% and 7.4% of the total wavelengths, respectively [22]. In the adulteration modeling of camellia seed oil, 25 characteristic wavelengths were selected via the CARS method, accounting for 2.49% of the total wavelengths [30]. After the characteristic wavelengths selection, the amount of data was greatly reduced, which reduced the calculation amount and improved the calculation efficiency.

The results of quantitative AV prediction models of camellia seed oil established via PCR and PLSR which are based on characteristic wavelengths are shown in Table 4. From this table, it is clear that the correlation coefficient of the calibration set ($R_{c}^{2}$) and prediction set ($R_{p}^{2}$) of the AV model based on SPA-selected characteristic wavelengths had slightly improved, while the performance of those based on CARS-selected characteristic wavelengths slightly decreased. The correlation coefficients of the calibration set ($R_{c}^{2}$) and prediction set ($R_{p}^{2}$) of the AV model for (2Der-SPA-PCR) camellia seed oil established via PCR which was based on SPA-selected characteristic wavelengths were 0.9299 and 0.9167, respectively. Compared with the optimal AV model based on full wavelengths (2Der-PLSR), the $R_{c}^{2}$ and $R_{p}^{2}$ were increased by 0.65% and 0.69%, respectively. Although the correlation coefficients of the optimal model were not significantly improved, the reduction in computational complexity greatly improved the modeling speed and stability. The correlation coefficients of the calibration set ($R_{c}^{2}$) and prediction set ($R_{p}^{2}$) of the AV model for (2Der-SPA-PLSR) camellia seed oil established using PLSR which was based on SPA-selected characteristic wavelengths were 0.9498 and 0.9341, respectively. Compared with the optimal AV model based on full wavelengths (2Der-PLSR), the $R_{c}^{2}$ and $R_{p}^{2}$ were increased by 2.64% and 2.43%, respectively. The optimal model for the hyperspectral moisture prediction of soybean was Normalize-SPA-PCR, with $R_{c}^{2}$ and $R_{p}^{2}$ at 0.9746 and 0.9778, respectively [22]. The optimal model for the adulteration amount of vegetable oil in camellia seed oil was CARS-PLSR, and the $R_{c}^{2}$ of the prediction set of the adulteration amount models for different vegetable oils ranged from 0.928 to 0.980 [30]. These studies show that the characteristic wavelengths selection could eliminate redundant wavelength variables, reduce the modeling computation, and improve the prediction ability of the model.

**Figure 6:**
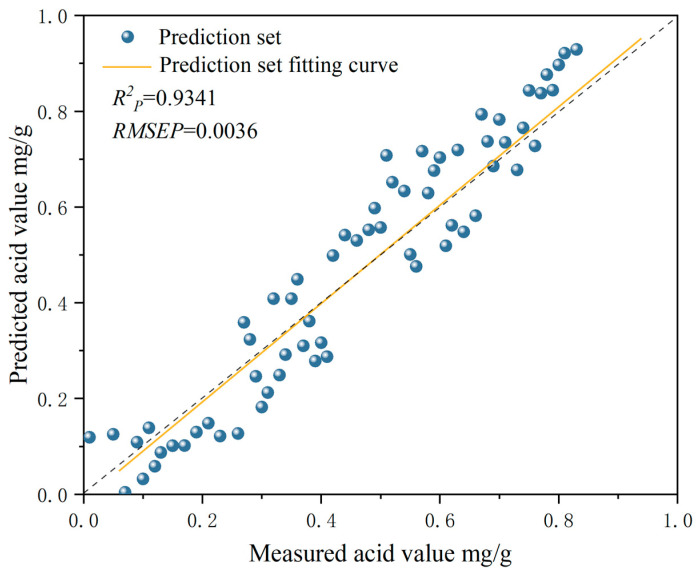
Correlations between measured and predicted acid value of camellia seed oil using the 2Der-SPA-PLSR model.

In summary, the results of the AV model for camellia seed oil based on characteristic wavelengths indicated that the PLSR model established after SPA selection (2Der-SPA-PLSR) had the best predictive performance. The prediction results of the 2Der-SPA-PLSR model is shown in Figure 6. It can be seen that the predicted acid value of camellia seed oil was highly correlated with the actual measured acid value. The correlation coefficient $R_{p}^{2}$ between the measured and predicted AV was 0.9341. The RMSEP was 0.0036, which indicated the good reliability of the prediction 2Der-SPA-PLSR model. It was concluded that the 2Der-SPA-PLSR model had good robustness and could accurately predict the AV of camellia seed oil.

### 3.5. Quantitative Modeling and Analysis of AV of Camellia Seed Oil Fusing Spectral and Image Features

**Figure 7:**
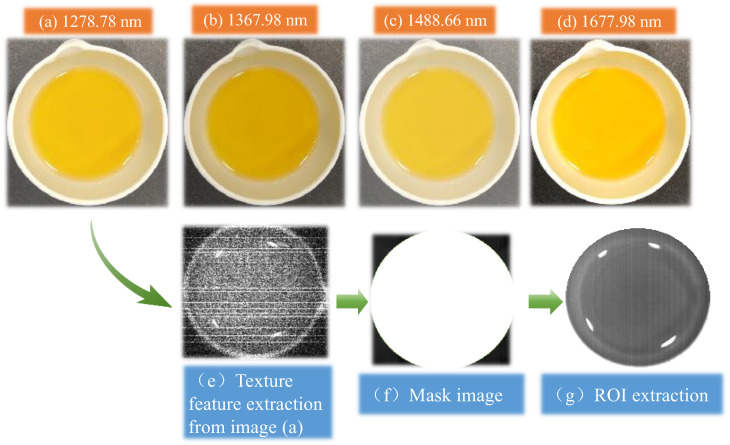
Hyperspectral image of No. 99 camellia seed oil sample at characteristic wavelengths and GLCM texture image.

According to the research results in Section 3.4, four characteristic wavelengths were selected using the SPA method. Combined with the hyperspectral images at characteristic wavelengths and spectral information, the quantitative AV prediction model of camellia seed oil fusing spectral and image features was established. Figure 7a–d are the hyperspectral images of No. 99 camellia seed oil sample at four characteristic wavelengths. GLCM algorithm was employed to extract the image features from the characteristic wavelength image. As an example, Figure 7e shows the result of texture feature extraction from Figure 7a. Texture features were extracted from hyperspectral images in four directions (0°, 45°, 90°, 135°) via the GLCM method, where texture features were described using four parameters: inverse different moment $f_{1}$, angular second moment $f_{2}$, entropy $f_{3}$, and correlation index $f_{4}$. The extracted image texture features were sequentially fused with spectral information to obtain a new set of feature vectors, and a quantitative AV prediction model for camellia seed oil was established.

The results of the quantitative AV prediction models of camellia seed oil established fusing spectral and image features are shown in Table 5. The optimal model for fusing spectral and image features was 2Der-SPA-GLCM-PLSR, and the correlation coefficient of the calibration set ($R_{c}^{2}$) and the correlation coefficient of the prediction set ($R_{p}^{2}$) were 0.9698 and 0.9581, respectively. Compared with the optimal AV model based on characteristic wavelengths (2Der-SPA-PLSR), the $R_{c}^{2}$ and $R_{p}^{2}$ were increased by 2.01% and 2.40%, respectively. Compared with the optimal AV model based on full wavelengths (2Der-PLSR), the $R_{c}^{2}$ and $R_{p}^{2}$ were increased by 4.64% and 4.83%, respectively. The results showed that the fusing of spectral and image features further improved the predictive performance of the AV model for camellia seed oil. The GLCM algorithm extracted effective information from characteristic wavelength images, enabling the established model to more accurately predict the AV of camellia seed oil. Most studies have reported that the selection of characteristic wavelengths and the fusion of spectral and image features could effectively improve the prediction performance of the model [36,37,38]. Bin L et al. established the optimal PLS-DA model for the damage degree discrimination of yellow peach via combining the characteristic spectral information with image features, and the prediction accuracy of the model for mild, moderate, and severe damage of yellow peach was 95%, 90%, and 95%, respectively [36]. Based on the fusing of spectral and image features, Cuiling L et al. conducted a rapid and nondestructive identification of Yingde black tea, and the research showed that the accuracy of the calibration set and prediction set of the optimal CARS-GLCM-GA-SVM model was 98.00% and 96.67%, respectively. Compared with the model CARS-GA-SVM without fusing image features, the accuracy of the calibration set and prediction set was improved by 11.34% and 18.37, respectively [38].

**Figure 8:**
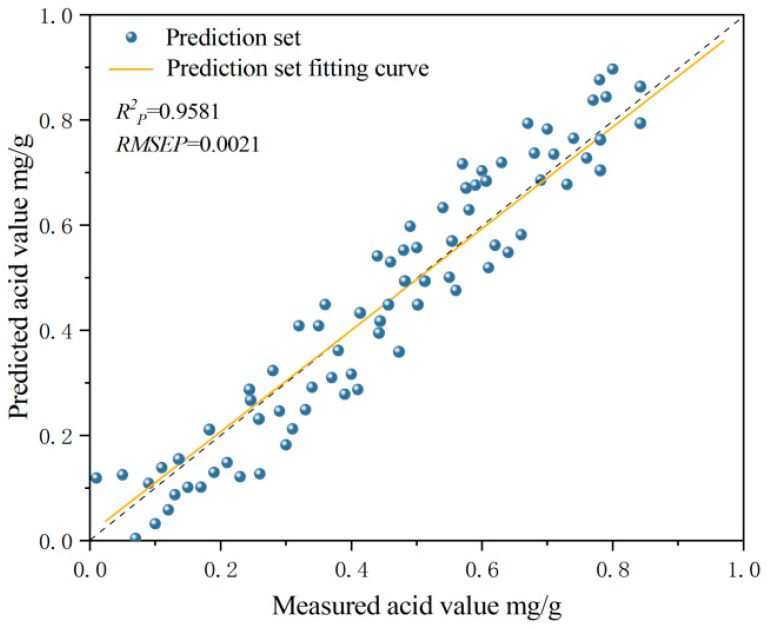
Correlations between measured and predicted acid value of camellia seed oil using 2Der-SPA-GLCM-PLSR model.

The prediction results of the 2Der-SPA-GLCM-PLSR model are shown in Figure 8. The correlation coefficient between the measured and predicted AV was 0.9581. The RMSEP was 0.0021, which indicated the good reliability of the prediction 2Der-SPA-GLCM-PLSR model. According to the prediction results of the 2Der-SPA-GLCM-PLSR model, the predicted acid value of camellia seed oil was highly correlated with the actual measured acid value. The 2Der-SPA-GLCM-PLSR model had good robustness and could be used for actual prediction.

### 3.6. Comparison of Modeling Results of Camellia Seed Oil AV

**Figure 9:**
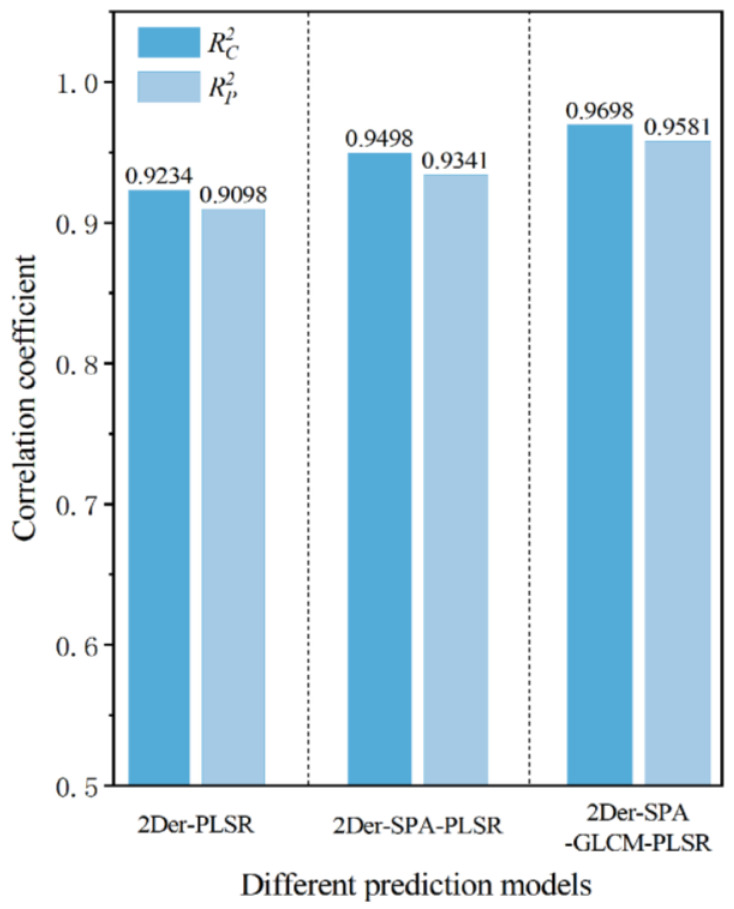
The comparison of different prediction models for AV.

The quantitative AV prediction models for camellia seed oil based on hyperspectral full wavelengths, characteristic wavelengths, and fusing spectral and image features were established in this study. The performance comparison of different models is shown in Figure 9. The optimal model for AV prediction based on hyperspectral full wavelengths was 2Der-PLSR; the correlation coefficient of the calibration set ($R_{c}^{2}$) was 0.9234 and the root mean square error of the calibration set (RMSEC) was 0.0865, while the correlation coefficient of the prediction set ($R_{p}^{2}$) was 0.9098 and the root mean square error of the prediction set (RMSEP) was 0.0731, respectively. The optimal model for AV prediction based on characteristic wavelengths was 2Der-SPA-PLSR, the correlation coefficient of the calibration set ($R_{c}^{2}$) was 0.9498 and the root mean square error of the calibration set (RMSEC) was 0.0057, while the correlation coefficient of the prediction set ($R_{p}^{2}$) was 0.9341 and the root mean square error of the prediction set (RMSEP) was 0.0036, respectively. The optimal model for AV prediction fusing spectral and image features was 2Der-SPA-GLCM-PLSR, the correlation coefficient of the calibration set ($R_{c}^{2}$) was 0.9698 and the root mean square error of the calibration set (RMSEC) was 0.0011, while the correlation coefficient of the prediction set ($R_{p}^{2}$) was 0.9581 and the root mean square error of the prediction set (RMSEP) was 0.0021, respectively. It can be seen that after spectral preprocessing, characteristic wavelengths selection, and fusing spectral and image features, the correlation coefficient of the optimal model for AV prediction was continuously improved, and the root mean square error was continuously decreased.

## 4. Conclusions

Taking camellia seed oil as the research object, combining hyperspectral imaging technology and chemometrics, the quantitative AV prediction models for camellia seed oil based on hyperspectral full wavelengths, characteristic wavelengths, and fusing spectral and image features were established in this study. The results demonstrated that the maximum, minimum, and the average AV of camellia seed oil were 1.59 mg/g, 0.32 mg/g, and 0.70 mg/g, respectively. The hyperspectral data of camellia seed oil effectively obtained the information needed for AV quantitative modeling. The proportion of characteristic wavelengths selected using SPA in the full wavelengths was 0.78%, and the amount of data was greatly reduced, which reduced the calculation amount and improved the calculation efficiency. By comparing the performance of various quantitative AV prediction models, it could be seen that after spectral preprocessing, characteristic wavelengths selection, and fusing spectral and image features, the correlation coefficient of the optimal model for AV prediction was continuously increased, and the root mean square error was continuously decreased. The 2Der-SPA-GLCM-PLSR model fusing spectral and image features stood out as the optimal choice for AV prediction of camellia seed oil. The $R_{c}^{2}$ and the RMSEC of the calibration set were 0.9698 and 0.0011, respectively, and the $R_{p}^{2}$ and the RMSEP of prediction set were 0.9581 and 0.0021, respectively. In conclusion, hyperspectral imaging technology could achieve high-precision quantitative prediction of the AV of camellia seed oil, and the results of this study could provide a theoretical foundation and data support for online and rapid detection of the acid value of food.

## Figures and Tables

**Table 1:** Determination results of acid value of camellia seed oil.

Sample	Sample Size	Maximum AV mg/g	Minimum AV mg/g	Average AV mg/g	Standard Deviation mg/g	Relative Standard Deviation%
All	153	1.59	0.32	0.70	0.33	46.64
Calibration set	92	1.58	0.31	0.68	0.32	47.92
Prediction set	61	1.59	0.32	0.70	0.33	46.45

**Table 2:** AV prediction models of camellia seed oil based on full wavelengths after different preprocessing.

Modeling Method	Preprocessing Method	$R_{c}^{2}$	RMSEC	$R_{p}^{2}$	RMSEP
PCR	None	0.7439	0.1790	0.6962	0.1651
	Normalize	0.8588	0.1119	0.8236	0.1477
	1Der	0.7063	0.1544	0.6265	0.1865
	2Der	0.9198	0.0892	0.9087	0.0844
	Baseline	0.8230	0.1134	0.7874	0.1356
	SNV	0.7945	0.0911	0.7854	0.0998
	SG	0.8493	0.1176	0.8173	0.1247
PLSR	None	0.7312	0.1480	0.6838	0.1448
	Normalize	0.7202	0.1770	0.6717	0.1832
	1Der	0.8678	0.1178	0.7531	0.1643
	2Der	0.9234	0.0865	0.9098	0.0731
	Baseline	0.8105	0.1380	0.7693	0.1524
	SNV	0.7123	0.1732	0.6594	0.1913
	SG	0.7809	0.1580	0.7345	0.1644

**Table 3:** The results of characteristic wavelengths selection via SPA and CARS.

Method	Wavelength/Number	Characteristic Wavelengths/nm	Proportion of Full Wavelengths/%
SPA	4	1278.78, 1367.98, 1488.66, 1677.98	0.78%
CARS	11	991.22, 1000.01, 1009.55, 1137.80, 1278.78, 1312.77, 1367.98, 1488.66, 1598.12, 1677.98, 1698.12	2.15%

**Table 4:** AV prediction models of camellia seed oil based on characteristic wavelengths.

Modeling Method	Wavelength Selection Method	Input Wavelength Number	$R_{c}^{2}$	RMSEC	$R_{p}^{2}$	RMSEP
PCR	None	512	0.9198	0.0892	0.9087	0.0844
	SPA	4	0.9299	0.0324	0.9167	0.0355
	CARS	11	0.8320	0.0641	0.8327	0.0505
PLSR	None	512	0.9234	0.0865	0.9098	0.0731
	SPA	4	0.9498	0.0057	0.9341	0.0036
	CARS	11	0.8979	0.0043	0.8478	0.0033

**Table 5:** AV prediction models of camellia seed oil fusing spectral and image features.

Modeling Method	$R_{c}^{2}$	RMSEC	$R_{p}^{2}$	RMSEP
PCR	0.9599	0.0034	0.9534	0.0065
PLSR	0.9698	0.0011	0.9581	0.0021

## Data Availability

The original contributions presented in the study are included in the article, further inquiries can be directed to the corresponding author.
